# Determinants of sexually transmitted infection-related care-seeking behavior among reproductive-age women in sub-Saharan Africa: A multilevel analysis

**DOI:** 10.1371/journal.pone.0331781

**Published:** 2025-09-10

**Authors:** Agazhe Aemro, Mohammed Seid Ali, Alebachew Ferede Zegeye, Belayneh Shetie Workneh, Gebreeyesus Abera Zeleke, Enyew Getaneh Mekonen, Tadesse Tarik Tamir, Mulugeta Wassie, Bewuketu Terefe, Berhan Tekeba

**Affiliations:** 1 Department of Medical Nursing, School of Nursing, College of Medicine and Health Sciences, University of Gondar, Gondar, Ethiopia; 2 Department of Pediatrics and Child Health Nursing, School of Nursing, College of Medicine and Health Sciences, University of Gondar, Gondar, Ethiopia; 3 Department of Emergency and Critical Care Nursing, School of Nursing, College of Medicine and Health Sciences, University of Gondar, Gondar, Ethiopia; 4 Department of Surgical Nursing, School of Nursing, College of Medicine and Health Sciences, University of Gondar, Gondar, Ethiopia; 5 Department of Community Health Nursing, School of Nursing, College of Medicine and Health Sciences, University of Gondar, Gondar, Ethiopia; Hawassa University College of Medicine and Health Sciences, ETHIOPIA

## Abstract

**Background:**

Sexually transmitted infections (STIs) remain a major public health threats in sub-Saharan Africa (SSA). Delay in seeking care is a significant barrier for the prevention and control of STIs. This study aimed to assess the proportion of women seeking STI-related care and its determinants in SSA.

**Method:**

This study was conducted using Demographic and Health Survey (DHS) data from eleven SSA countries between 2017/18 and 2022/23. It included 47,924 reproductive-age women who reported having STIs or STI symptoms. A multilevel logistic regression model was fitted to identify factors associated with STI-related care-seeking behavior. The strength of association was estimated using adjusted odds ratios (AORs) with 95% confidence intervals (CIs). Finally, variables with a p-value of <0.05 were considered statistically significant predictors.

**Result:**

In this study, the overall proportion of women seeking STI-related care was 62.2%. Younger age (AOR = 0.76; 95% CI: 0.70, 0.82), higher education level (AOR = 1.20; 95% CI: 1.13, 1.28), higher wealth index (AOR = 1.58; 95% CI: 1.48, 1.69), having >1 sexual partner (AOR = 1.55; 95% CI: 1.37, 1.75), ever heard of STIs (AOR = 2.16; 95% CI: 1.89, 2.47), asking a husband to use a condom (AOR = 2.10; 95% CI: 1.99, 2.22), being pregnant (AOR = 1.29; 95% CI: 1.21, 1.38), media exposure (AOR = 1.20; 95% CI: 1.14, 1.27), residing in West Africa (AOR = 1.51; 95% CI: 1.41, 1.61), being interviewed in 2019 (AOR = 2.27; 95% CI: 2.13, 2.42), and living in a community with low poverty levels (AOR = 1.23; 95% CI: 1.08, 1.40) were identified as significant predictors of STI-related care-seeking behavior.

**Conclusion:**

This study revealed that a significant proportion of women did not seek STI-related care. Therefore, national strategies and policies should be implemented to address barriers to seeking care.

## Introduction

Sexually transmitted infections (STIs) are a serious public health threat [1,2], primarily transmitted through sexual activity and caused by bacteria, parasites, or viruses. The most common STIs include gonorrhea, chlamydia, syphilis, trichomoniasis, herpes simplex virus infection, human papillomavirus infection, and human immunodeficiency virus infection [3–5]. Since many STIs are asymptomatic, individuals may unknowingly spread infections to their sexual partners. Fortunately, many STIs are curable, and for those that cannot yet be cured, medications are available to effectively reduce symptoms and minimize the risk of transmission [6]. However, the effectiveness of STI prevention and treatment outcomes depends on individuals’ care-seeking behavior [2].

STI-related care-seeking behavior refers to the actions individuals take to address STI-related health concerns [7]. This is crucial for controlling the spread of STIs and improving public health outcomes [2]. It comprises recognizing symptoms, seeking medical advice, undergoing testing, obtaining treatment, and engaging in preventive measures. Several factors influence care-seeking behavior, including personal, social, economic, and healthcare system-related factors [8–11]. Previous studies have identified key determinants of STI-related care-seeking behavior, such as age, educational status, marital status, place of residence, social stigma, lack of awareness, financial constraints, cultural barriers, attitudes toward sexual health, number of sexual partners, and access to healthcare services [7,8,12–15]. Addressing these multifaceted factors through effective health education and barrier reduction strategies is essential for promoting positive STI-related care-seeking behaviors [4].

The burden of STIs is disproportionately high in low- and middle-income countries, including sub-Saharan Africa (SSA). Since STIs primarily affect the productive-age population, SSA faces a significant loss of its workforce due to STI-related health complications [16]. Moreover, reproductive-age women are particularly vulnerable to STIs due to biological, social, and healthcare-related factors. Anatomically, women are more susceptible to STIs and face a higher risk of severe reproductive health issues, including pelvic inflammatory disease, ectopic pregnancy, infertility, preterm labor, low birth weight, and increased infant mortality. Additionally, cultural perceptions linking STIs with promiscuity contribute to fear of judgment and hesitancy in seeking care. Many women also lack financial independence and face challenges accessing healthcare facilities, further delaying timely care and treatment [17–19].

Due to the asymptomatic nature of many STIs, women often seek care too late, posing a major barrier to STI prevention and control among the reproductive-age population [8,15,18]. This issue is particularly evident in SSA, where lower socioeconomic status, limited female empowerment in healthcare decision-making, and societal stigma remain significant concerns. The COVID-19 pandemic further impacted STI care and treatment. During the pandemic, fear of infection, lockdown measures, and disruptions in healthcare services led to reduced access to STI clinics, testing, and treatment [20]. Despite the high burden of STIs in SSA, recent evidence on STI-related healthcare-seeking behavior remains limited. Therefore, this study aimed to assess the proportion of reproductive-age women seeking STI-related care and its determinants in sub-Saharan Africa.

## Methods

### Data source and study design

A cross-sectional analysis was conducted using the most recent Demographic and Health Survey (DHS) datasets from 11 sub-Saharan African (SSA) countries, issued between 2017/18 and 2022/23. The DHS surveys are designed to collect cross-country comparable data on marriage, fertility, mortality, family planning, reproductive health, child health, nutrition, and HIV/AIDS. Due to the subject matter of the survey, women of reproductive age (15–49 years) are the primary focus. Women eligible for individual interviews are identified through selected households within the survey sample. Additionally, the DHS program collects data on various health indicators, such as immunization coverage, Vitamin A supplementation, antenatal care coverage, fertility rate, early marriage, child mortality rate, infant mortality rate, maternal mortality ratio, and others [21].

SSA countries with available data on the outcome of interest within the specified time period were included in the study. From these countries, reproductive-age women who had an STI or symptoms of an STI within the previous 12 months were the target population. Accordingly, the Individual Record (IR) file from each included SSA country served as the dataset for this study. After pooling the datasets together through appending, a multilevel regression analysis model was fitted.

### Study population and sampling technique

**Fig 1:**
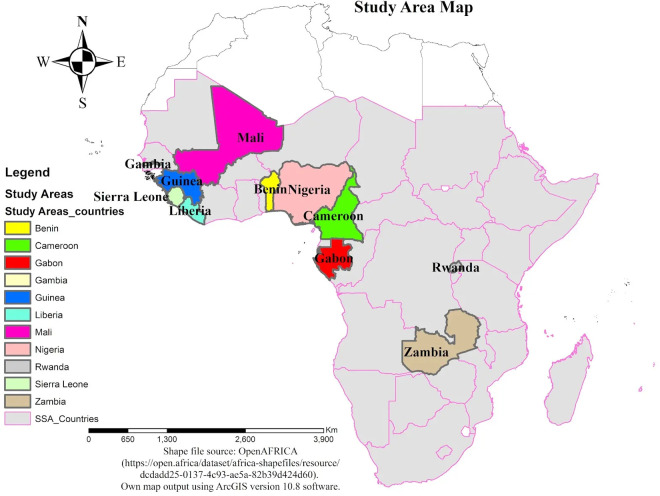
Study area map to show included Sub-Saharan Africa countries to assess STI-related care-seeking behavior (Shapefile source: Open AFRICA, https://open.africa/dataset/africa-shapefiles/resource/dcdadd25-0137-4c93-ae5a-82b39d424d60; Map created by the authors using ArcGIS version 10.8).

The study used the most recent IR dataset from 11 SSA countries. Within the specified period (2017/18–2022/23), DHS data from 16 SSA countries were accessible via the DHS website upon completing a brief form outlining the study’s objectives and methods. These countries included Benin, Cameroon, Gabon, Gambia, Guinea, Kenya, Liberia, Mali, Malawi, Mozambique, Nigeria, Rwanda, Sierra Leone, Senegal, Tanzania, Zambia, and Ghana. However, data from five countries (Kenya, Mozambique, Senegal, Tanzania, and Ghana) lacked records on the outcome of interest for this study, leading to their exclusion from the final analysis (Fig 1).

The DHS survey employs a two stage cluster sampling technique to select respondents. This study included a total weighted sample of 47,924 reproductive-age women who reported having an STI or STI symptoms and had recorded data on the outcome of interest (i.e., seeking advice or treatment for their last STI infection) (Table 1).

**Table 1:** Description of study samples for the assessment of STI-related care-seeking behavior among reproductive-age women in sub-Saharan Africa.

Country	Year of survey	Weighted frequency (n)	Percentage (%)
Benin	2017/18	3,360	7.01
Cameroon	2018	6,279	13.10
Gabon	2019 - 2021	2,814	5.87
Gambia	2019/20	3,255	6.79
Guinea	2018	3,056	6.38
Liberia	2019/20	6,848	14.29
Mali	2018	5,586	11.66
Nigeria	2018	10,312	21.52
Rwanda	2019/20	2,792	5.83
Sierra Leone	2019	2,994	6.25
Zambia	2018/19	628	1.31
Total		47,924	100

## Study variables

### Dependent/outcome variable

The outcome variable for this study was the percentage of women who sought care (advice or treatment) for an STI or STI symptoms in the past 12 months. Based on the DHS report, participants were first identified as having an STI or STI symptoms if they answered “Yes” to at least one of the following questions: a) Had any STI in the last 12 months; b) Had genital sores or ulcers in the last 12 months; c) Had genital discharge in the last 12 months. Women who answered “Yes” to at least one of these questions were further asked whether they sought advice or treatment for the condition. A woman was classified as having sought advice or treatment for an STI if she answered “Yes” to the question: “Have you sought advice or treatment for an STI infection in the last 12 months?” Additionally, according to DHS guidelines, missing values were assumed to indicate that no advice or treatment was sought regarding STIs or STI symptoms [21].

### Explanatory variables

Following an extensive literature review, 21 explanatory variables were identified and grouped into individual-level and community-level factors.

**Individual-Level Variables:** Age of women [2,11], women’s educational status [7,14], women’s working status, residence [7], marital status [2,9], household wealth index [11,14], health insurance coverage, number of sexual partner including spouse [7], distance from health facility [7,11], ever heard of STIs, recent sexual activity in the last 4 weeks, wife justified asking her husband to use condom if he has STI, cohabitation duration, current pregnancy status [7,14], cigarette smoking, media exposure status [11], year of interview, and Country region.

**Women’s working status:** is derived from the question “Respondent currently working?” If the respondent said ‘Yes’, the women is said to be currently working, otherwise not.

**Household wealth index:** in the DHS dataset, this variable is already calculated and labeled in to five-level Likert scales (poorest, poorer, middle, richer, and richest).

**Community-level variables:** Community-level variables were computed by aggregating individual-level data at the cluster level; residence, community level poverty (aggregated from household wealth index), community illiteracy (aggregated from maternal education), and community media exposure (aggregated from frequency of watching television, reading newspaper, and listening radio).

### Statistical analysis

After identifying, cleaning, and recoding of variables based on literatures, the authors pooled the DHS data from eleven SSA countries. STATA version 17 statistical software was used for recoding and analysis of the data. The weighting sample was applied to address issues related to under or over-sampling, and to establish its representativeness. Descriptive statistics were described by using frequencies, percentages, means, and standard deviations (SD). The results was presented using texts, tables, and figures. Since the DHS data was based on clusters, the assumptions of independent observations and equal variance across clusters could not meet. So, using of models other than a standard logistic regression model is important. Based on this, the authors computed a multilevel logistic regression analysis in order to determine the factors associated with STI-related care-seeking behavior. First, we computed bi-variable multilevel logistic regression, and those variables with p-value of ≤ 0.2 were candidate for multivariate analysis.

### Random effects and model fitness

After selecting variables for multilevel analysis, four models were fitted: Null model (outcome variable only) assesses how much variation in STI-related care-seeking behavior is due to group-level factors before adding predictors. **Model I** (individual-level variables with the outcome variable): adds individual-level factors to assess whether individual predictors explain part of the between-group variation of STI-related care-seeking. **Model II** (community-level variables with the outcome variable): adds community-level factors to evaluate how much of the remaining between-group variation in STI-related care-seeking is explained by these factors. **Model III** (both individual and community-level variables with the outcome variable): tests whether the effect of individual-level factors on STI-related care-seeking behavior varies depending on the community-level context.

The intra-class correlation coefficient (ICC) and proportional change in variance (PCV) were used to assess the random effect or measures of variation of the STI-related care-seeking behavior across communities or clusters. PCV quantifies the reduction in variance of STI-related care-seeking behavior at a specific level when a predictor variable is added to a multilevel model. Whereas, ICC measures the proportion of total variance in STI-related care-seeking behavior that is due to differences between clusters. Model comparison was performed by using the deviance test, and the model with the lowest deviance was considered as the best-fitted model. Finally, by computing the best fitted model, variables with p-value of <0.05 with their corresponding 95% confidence interval (CI) were considered as statistically significant factors of STI-related care-seeking behavior. An Adjusted Odds Ratio (AOR) was also computed to show the strength of association between the explanatory and outcome variable. To address confounders, different strategies were implemented at both the design and analysis phase. During the design phase, we used survey weights to ensure a representative estimate. During the analysis phase, we used survey-weighed regression models to adjust for confounders.

### Ethics approval and consent to participate

This study was conducted by using the DHS datasets, which are publicly available at https://www.dhsprogram.com/data/available-datasets.cfm. Once registered and granted access permission, researchers can download the datasets from the required countries. Therefore, obtaining informed consent from participants or ethics committee approval is not necessary.

## Results

### Individual and reproductive health characteristics of the respondents

In this study, a total of 47,924 reproductive-age women who had an STI or symptoms of an STI were included in the synthesis of the results. Of these, nearly 38% were aged 25–34 years. The mean age of the respondents was 29.63 ± 8.53 years, with a minimum and maximum age of 15 and 49 years, respectively. Similarly, 40% of the respondents had attained at least a secondary-level education. Around 55% of the participants resided in urban areas. The majority of the women (87.82%) in this study did not have health insurance coverage, and almost all (97%) had heard of STIs. Additionally, approximately 71% of the respondents had media exposure (Table 2).

**Table 2:** Individual, Household, and Reproductive Health Characteristics of Women Aged 15–49 Years in Sub-Saharan Africa (N = 47,924).

Variables	Categories	Frequency (n)	Percentage (%)
Women’s current age	15 - 24 years	15,408	32.15
	25 - 34 years	18,167	37.91
	35 - 49 years	14,349	29.94
Women currently working	Yes	31,540	65.81
	No	16,384	34.19
Women’s education level	No education	17,988	37.53
	Primary education	10,730	22.39
	Secondary & above	19,206	40.08
Residence	Rural	26,544	55.39
	Urban	21,380	44.61
Marital status	Never in union	9,140	19.07
	Currently in union	35,066	73.17
	Formerly in union	3,718	7.76
Household wealth index	Poor	19,197	40.06
	Middle	9,762	20.37
	Rich	18,965	39.57
Covered by health insurance	Yes	5,838	12.18
	No	42,086	87.82
Number of sex partners in the last 12 months	Zero	4,807	10.03
	One	40,374	84.25
	Two or more	2,743	5.72
Distance to health facility	Big problem	17,232	35.96
	Not a big problem	30,692	64.04
Ever heard of STIs	Yes	46,480	96.99
	No	1,444	3.01
Recent sexual activity	Active	30,993	64.67
	Not active	16,931	35.33
Wife justified asking husband to use condom if he has STI	Yes	38,684	80.72
	No	9,240	19.28
Cohabitation duration	< 5 years	17,974	37.51
	5-9 years	9,008	18.80
	10-19 years	13,397	27.95
	>19 years	7,545	15.74
Currently pregnant	Yes	5,446	11.36
	No	42,478	88.64
Smoking cigarettes	Yes	669	1.40
	No	47,255	98.60
Had media exposure	Yes	34,213	71.39
	No	13,708	28.61
Community-level poverty status	Low	24,766	51.68
	High	23,158	48.32
Community-level Illiteracy status	Low	25,765	53.76
	High	22,159	46.24
Community-level media exposure status	Low	22,095	46.10
	High	25,829	53.90
Region of study	West Africa	35,411	73.89
	Central Africa	9,093	18.97
	East Africa	3,420	7.14
Year of interview	2018	29,196	60.92
	2019	8,594	17.93
	2020	8,910	18.59
	2021	1,224	2.55

### Model fitness and random effect analysis

The results of this study revealed that Model III was the best-fitting model (the model with the lowest deviance) for identifying factors associated with STI-related care-seeking behavior among reproductive-age women in sub-Saharan Africa. In the Null Model for example, the ICC was 19.92%, meaning 19.92% of the variation in STI-related care-seeking behavior is due to group-level factors, justifying the use of a multilevel model. In the final Model, the ICC was 14.6%, meaning, 14.6% of the variation in STI-related care-seeking behavior among reproductive-age women is due to combination of Individual- and community-level factors (cross-level interactions). Similarly, the PCV of Model III indicated that 31.5% of the variation in STI-related care-seeking behavior was attributable to both individual- and community-level variables included in the model (Table 3).

**Table 3:** Model Fitness and Random Effect Analysis for STI-Related Care-Seeking Behavior among Reproductive-Age Women in Sub-Saharan African Countries.

Parameter	Null model	Model I	Model II	Model III
Log likelihood ratio (LLR)	−30842.726	−28671.628	−30310.163	−28659.146
Deviance = −2*LLR	61685.452	57343.256	60620.326	57318.292
Variance of the cluster (VC)	0.8182231	0.57543	0.6508445	0.5605184
ICC = VC/(VC + 3.29)×100%	19.92%	14.9%	16.5%	14.6%
$PCV=\frac{Vnull-VC}{Vnull}\times 100\%$	Reference	29.7%	20.5%	31.5%

### Proportion of women who sought care for STIs in sub-Saharan Africa

**Fig 2:**
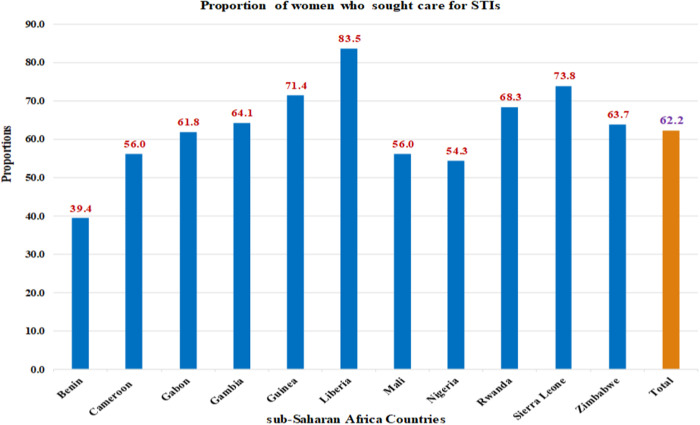
Proportion of Women Who Sought Care for STIs in Sub-Saharan Africa.

In this study, the overall proportion of reproductive-age women in sub-Saharan Africa who sought care for STIs was 62.2% (95% CI: 61.8%–62.6%). The lowest proportion (39.4%) and highest proportion (83.5%) was reported in Benin and Liberia, respectively (Fig 2).

**Fig 3:**
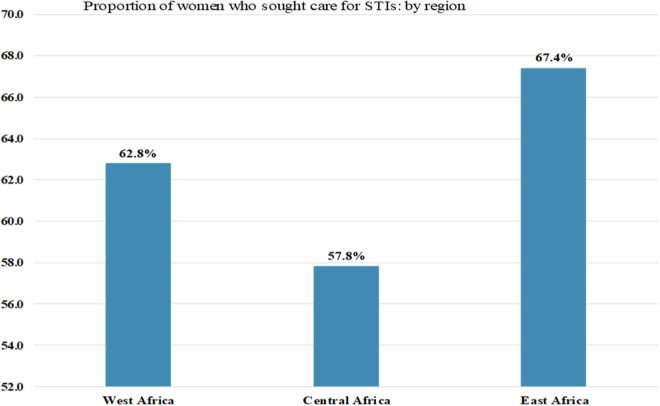
Proportion of Women Who Sought Care for STIs among Reproductive-Age Women by Region in Sub-Saharan Africa.

Additionally, we estimated the proportion of women who sought care for STIs by region. Hence, the highest proportion was identified in East Africa (67.4%) (Fig 3).

### Determinants of STI-related care-seeking behavior among reproductive-age women in sub-Saharan Africa

After applying multivariable multilevel regression analysis, several statistically significant determinants of STI-related care-seeking behavior among reproductive-age women in SSA were identified. These included women’s age, education level, residence, marital status, household wealth index, health insurance coverage, number of sex partners, distance to a health facility, awareness of STIs, justification for condom use, pregnancy status, media exposure, region, year of the interview, and community-level poverty status. Younger women were 24% less likely to seek care for STIs or STI symptoms compared to older women (AOR = 0.76; 95% CI: 0.70–0.82). Similarly, women with at least secondary-level education were 20% more likely to seek STI-related care than those with no formal education (AOR = 1.20; 95% CI: 1.13–1.28). Women in the highest wealth index category were 58% more likely to seek care compared to those in the poorest category (AOR = 1.58; 95% CI: 1.48–1.69). Women with two or more sexual partners were 55% more likely to seek care compared to those with no partners (AOR = 1.55; 95% CI: 1.37–1.75). Women who had heard of STIs were more than twice as likely to seek STI-related care compared to those who had not (AOR = 2.16; 95% CI: 1.89–2.47). Women who justified asking their husband to use a condom if he had STI were twice as likely to seek care (AOR = 2.10; 95% CI: 1.99–2.22). Pregnant women were 29% more likely to seek STI-related care than non-pregnant (AOR = 1.29; 95% CI: 1.21–1.38). Women exposed to media were 20% more likely to seek care (AOR = 1.20; 95% CI: 1.14–1.27). Women in West Africa (AOR = 1.51; 95% CI: 1.41–1.61), those interviewed in 2019 (AOR = 2.27; 95% CI: 2.13–2.42), and those from low community-level poverty areas (AOR = 1.23; 95% CI: 1.08–1.40) had higher odds of seeking care for STIs (Table 4).

**Table 4:** Determinants of STI-related Care-Seeking Behavior among Reproductive-Age Women in sub-Saharan Africa Using the Best-Fitting Model.

Variables	Categories	Care seek for STI		COR (95% CI)	AOR (95% CI)
		Yes	No		
Women’s current age	15 - 24 years	9,171	6,237	0.85 (0.81–0.89)	0.76 (0.70 - 0.82)**
	25 - 34 years	11,748	6,419	1.11 (1.06–1.16)	1.04 (0.97 - 1.11)
	35 - 49 years	8,889	5,460	1.00	1.00
Women currently working	Yes	19,917	11,623	1.10 (1.05–1.14)	1.12 (1.07 - 1.17)
	No	9,891	6,493	1.00	1.00
Women’s education level	No education	9,841	8,147	1.00	1.00
	1^0^ education	6,623	4,107	1.32 (1.25–1.40)	1.02 (0.96 - 1.08)
	2^0^ & above	13,344	5,862	1.95 (1.86–2.04)	1.20 (1.13 - 1.28)**
Residence	Rural	14,876	11,668	1.00	1.00
	Urban	14,932	6,448	2.08 (1.98–2.17)	1.17 (1.11 - 1.25)**
Marital status	Never in union	6,138	3,002	1.00	1.00
	Currently in union	21,156	13,910	0.76 (0.72 - 0.80)	0.96 (0.89 - 1.03)
	Formerly in union	2,514	1,204	1.07 (0.98 - 1.17)	1.23 (1.11 - 1.36)**
Household wealth index	Poor	10,312	8,885	1.00	1.00
	Middle	6,025	3,737	1.40 (1.33–1.48)	1.20 (1.13 - 1.27)**
	Rich	13,471	5,494	2.31 (2.20–2.43)	1.58 (1.48 - 1.69)**
Covered by health insurance	Yes	4,153	1,685	1.77 (1.66–1.89)	1.22 (1.12 - 1.33)**
	No	25,655	16,431	1.00	1.00
Number of sex partners in the last 12 months	Zero	2,759	2,048	1.00	1.00
	One	25,075	15,299	1.23 (1.14 - 1.30)	1.27 (1.17 - 1.37)**
	Two or more	1,974	769	1.86 (1.67 - 2.07)	1.55 (1.37 - 1.75)**
Distance to health facility	Big problem	9,918	7,314	1.00	1.00
	Not big problem	19,890	10,802	1.39 (1.33–1.45)	1.11 (1.06 - 1.16)**
Ever heard of STIs	Yes	29,376	17,104	4.16 (3.66–4.73)	2.16 (1.89 - 2.47)**
	No	432	1,012	1.00	1.00
Recent sexual activity	Active	19,415	11,578	1.04 (1.00–1.09)	1.03 (0.98 - 1.09)
	Not active	10,393	6,538	1.00	1.00
Wife asked husband to use condom	Yes	25,918	12,766	2.67 (2.54–2.81)	2.10 (1.99 - 2.22)**
	No	3,890	5,350	1.00	1.00
Cohabitation duration	< 5 years	11,497	6,477	1.00	1.00
	5 - 9 years	5,576	3,432	0.94 (0.89–0.99	1.02 (0.95 - 1.10)
	10 −19 years	8,273	5,124	0.93 (0.89–0.98)	0.98 (0.91 - 1.06)
	>19 years	4,462	3,083	0.84 (0.79–0.89)	0.95 (0.86 - 1.05)
Currently pregnant	Yes	3,471	1,975	1.10 (1.03–1.17)	1.29 (1.21 - 1.38)**
	No	26,337	16,141	1.00	1.00
Smoking cigarettes	Yes	412	200	1.48 (1.25–1.76)	1.15 (0.96 - 1.37)
	No	29,396	17,916	1.00	1.00
Had media exposure	Yes	22,446	11,767	1.67 (1.60–1.75)	1.20 (1.14 - 1.27)**
	No	7,359	6,349	1.00	1.00
Region of study	West Africa	22,243	13,168	1.14 (1.08–1.20)	1.51 (1.41 - 1.61)**
	Central Africa	5,259	3,834	1.00	1.00
	East Africa	2,306	1,114	1.38 (1.26–1.51)	1.08 (0.97 - 1.21)
Year of interview	2018	16,129	13,067	1.00	1.00
	2019	6,643	1,951	2.99 (2.81–3.17)	2.27 (2.13 - 2.42)**
	2020	6,283	2,627	1.94 (1.82–2.06)	1.56 (1.45 - 1.68)**
	2021	753	471	2.27 (1.93–2.66)	1.8 (1.51 - 2.16)**
Community-level poverty status	Low	16,437	8,329	1.93 (1.71–2.18)	1.23 (1.08 - 1.40)*
	High	13,371	9,787	1.00	1.00
Community-level Illiteracy status	Low	16,751	9,014	1.73 (1.53–1.96)	1.10 (0.97 - 1.25)
	High	13,057	9,102	1.00	1.00
Community-level media exposure	Low	12,818	9,277	1.00	1.00
	High	16,990	8,839	1.67 (1.47–1.88)	1.05 (0.93 - 1.20)

*significant at p-value <0.05; ** significant at p-value ≤0.001.

## Discussion

To control STIs, seeking care for STIs or STI symptoms is essential [2]. However, many women, particularly in sub-Saharan Africa, do not seek STI-related care. Additionally, delays in seeking care contribute to sustained transmission and a greater likelihood of adverse complications [3]. Therefore, assessing the proportion of women seeking STI-related care and identifying the barriers they face was crucial, which led us to conduct this research.

In this study, the overall proportion of reproductive-age women in sub-Saharan Africa who sought care for STIs was 62.2% (95% CI: 61.8% − 62.6%). This is lower than the proportions reported in studies conducted in India (77.4%), a previous study in SSA (66.1%), and Tanzania (64%). The study in Tanzania focused on women working in bars and hotels—settings with a higher risk of STI transmission. Due to higher risk of STI infection in these environments, individuals with STIs or STI symptoms might be more motivated to seek care, contributing to the higher percentage [22].

Conversely, the proportion of women who sought care for STIs or STI symptoms in our study is higher than in studies conducted in Ethiopia (33.3%, using DHS 2016), Bangladesh (50%), India (47.6%), Nigeria (55.7%), and East Africa (54.1%). The study in East Africa used DHS data from as early as 2008, which is significantly older than the data in our study [7]. Over time, generational attitudes toward health, including STI-related care, have improved, which may explain the higher proportion in our findings. Additionally, the smaller sample sizes in studies from Ethiopia (n = 474), Nigeria (n = 4997), and Bangladesh (n = 240) might have underestimated the proportion of women seeking STI-related care compared to our study [2,8,14].

Identifying and addressing barriers to STI-related care is essential to reducing delays or failure in seeking medical attention for STIs or STI symptoms. Thus, we analyzed DHS data to identify factors that positively or negatively affect STI-related care-seeking behavior among reproductive age women in SSA. This study revealed that younger women were 24% less likely to seek care for STIs or STI symptoms compared to older women. This finding aligns with previous studies conducted in Africa [7,16] and Bangladesh [2]. Compared to older women, younger women may lack knowledge about STIs and the importance of seeking care. Additionally, in many SSA countries, young women are not expected to be sexually active, which can lead to embarrassment and reluctance to seek care. Financial dependence also plays a role, as younger women may hesitate to seek care without financial assistance. This implies that younger individuals may continue engaging in sexual activity and perpetuate STI transmission within communities. Therefore, public health services must be youth-friendly and focus on educating young adults about sexual health, including STIs [9,23].

Similarly, women with higher levels of education were more likely to seek care for STIs or STI symptoms compared to those with no formal education. This finding is consistent with previous studies in Africa [7,14,16]. Education increases knowledge about health issues, and more educated women are more aware of STIs and their consequences. This emphasizes the need for public health strategies that integrate comprehensive sexual health education into school curricula and community programs to ensure equitable access to STI-related information [15,24]. Likewise, women with a higher wealth index were also more likely to seek STI-related care, a result consistent with studies from India [11] and previous studies in Africa [7,14,16]. Women from wealthier families face fewer financial barriers to healthcare, making it easier for them to seek STI-related care. This highlights the importance of public health policies that ensure STI services are affordable and accessible to all, regardless of socioeconomic status [17,19].

The number of sexual partners was another key determinant of care-seeking behavior. Women with two or more sexual partners were more likely to seek STI-related care, a finding consistent with studies conducted in East Africa [7,15]. Engaging in multiple sexual partnerships increases the risk of STI infection, making women in this category more aware of their risk and, therefore, more likely to seek care. This suggests public health strategies should focus on educating individuals with multiple partners about the importance of regular testing and early treatment [19]. Furthermore, women who had heard about STIs were more than twice as likely to seek STI-related care compared to those who had not. This aligns with a study conducted in Ethiopia [15]. Awareness of STIs—including their symptoms, transmission methods, prevention, and treatment—encourages women to seek medical care when necessary. This finding emphasizes the importance of public health campaigns that promote STI awareness, which could help reduce infection rates [24].

Women who justified asking their husbands to use condoms if they had an STI were also about twice as likely to seek care. The reason behind this could be that empowered women who can negotiate condom use may also be more proactive about seeking STI-related care, leading to earlier diagnosis and prevention of further transmission. This stresses the importance of promoting gender equality in sexual and reproductive health decision-making [25]. Pregnant women were nearly 30% more likely to seek care compared to non-pregnant women, consistent with studies conducted in East Africa [7,14]. Pregnant women are more concerned about their health due to potential adverse pregnancy outcomes for both themselves and their babies. Additionally, pregnancy often increases healthcare engagement, as prenatal care typically includes routine STI screening. This highlights the importance of pregnancy as a crucial time for STI-related care-seeking behavior, as it provides greater access to healthcare and intervention opportunities [17,18].

Media exposure was another significant determinant of STI-related care-seeking behavior. Women who had media exposure were more likely to seek STI-related care, consistent with previous studies in Africa [16]. Increased media exposure provides more opportunities to access information about STIs, making women more likely to seek medical care when needed. This highlights the positive impact of mass media on promoting health awareness in communities [26]. Likewise, the odds of seeking STI-related care were also about twice as high for women interviewed in 2019 compared to those interviewed in 2018. Although this difference is not supported by other studies, it may be attributed to variations in surveillance, data collection, and reporting methods. As a result, women with STIs or STI symptoms in 2019 may have been more likely to seek care [27]. Lastly, our study found that women in West Africa were 51% more likely to seek STI-related care (AOR = 1.51) compared to those in Central Africa. While no previous studies specifically support this finding, potential explanations include differences in societal norms, stigma around disclosing STIs, variations in healthcare system attributes, and disparities in geographic or financial access to healthcare services across regions. These differences may account for regional variations in STI related care-seeking behavior [10,13].

Although this study provides valuable insights into the barriers to STI-related care among reproductive-age women in SSA, it has some limitations. Only eleven SSA countries were included due to the unavailability of recent datasets for other countries, which may affect the generalizability of the findings. Additionally, due to missing data on partner-related variables among those who had not partner, these factors were therefore excluded. Finally, as this study relied on secondary data, important variables such as attitudes and knowledge about STIs were not included.

## Conclusion

Although the percentage of women seeking STI-related care in this study exceeds fifty percent, a significant proportion of women still did not seek STI-related care and continues to be a major public health concern in SSA. To improve STI-related care-seeking behavior, special attention should be given to younger women, those with lower educational levels, and those from lower socioeconomic backgrounds. Mass media campaigns and community health education programs should be used to raise awareness about the importance of seeking STI-related care. Furthermore, national strategies and policies should be implemented to address barriers to healthcare accessibility.

## Abbreviations

AOR: adjusted odds ratio
CI: confidence interval
COR: crude odds ratio
DHS: demographic and heath surveys
ICC: intra-class correlation coefficient
IR: individual record
LLR: log likelihood ratio
PCV: proportional change in variance
SD: standard deviation
SSA: sub-Saharan Africa
STIs: sexually transmitted infections
WHO: World Health Organization.
